# Tribendimidine: Mode of Action and nAChR Subtype Selectivity in *Ascaris* and *Oesophagostomum*

**DOI:** 10.1371/journal.pntd.0003495

**Published:** 2015-02-13

**Authors:** Alan P. Robertson, Sreekanth Puttachary, Samuel K. Buxton, Richard J. Martin

**Affiliations:** Department of Biomedical Sciences, Iowa State University, Ames, Iowa, United States of America; Swiss Tropical and Public Health Institute, SWITZERLAND

## Abstract

The cholinergic class of anthelmintic drugs is used for the control of parasitic nematodes. One of this class of drugs, tribendimidine (a symmetrical diamidine derivative, of amidantel), was developed in China for use in humans in the mid-1980s. It has a broader-spectrum anthelmintic action against soil-transmitted helminthiasis than other cholinergic anthelmintics, and is effective against hookworm, pinworms, roundworms, and *Strongyloides* and flatworm of humans. Although molecular studies on *C. elegans* suggest that tribendimidine is a cholinergic agonist that is selective for the same nematode muscle nAChR as levamisole, no direct electrophysiological observations in nematode parasites have been made to test this hypothesis. Also the hypothesis that levamisole and tribendimine act on the same receptor, does not explain why tribendimidine is effective against some nematode parasites when levamisole is not. Here we examine the effects of tribendimidine on the electrophysiology and contraction of *Ascaris suum* body muscle and show that tribendimidine produces depolarization antagonized by the nicotinic antagonist mecamylamine, and that tribendimidine is an agonist of muscle nAChRs of parasitic nematodes. Further pharmacological characterization of the nAChRs activated by tribendimidine in our *Ascaris* muscle contraction assay shows that tribendimidine is not selective for the same receptor subtypes as levamisole, and that tribendimidine is more selective for the B-subtype than the L-subtype of nAChR. In addition, larval migration inhibition assays with levamisole-resistant *Oesophagostomum dentatum* isolates show that tribendimidine is as active on a levamisole-resistant isolate as on a levamisole-sensitive isolate, suggesting that the selectivity for levamisole and tribendimidine is not the same. It is concluded that tribendimidine can activate a different population of nematode parasite nAChRs than levamisole, and is more like bephenium. The different nAChR subtype selectivity of tribendimidine may explain why the spectrum of action of tribendimidine is different to that of other cholinergic anthelmintics like levamisole.

## Author Summary

Nematode parasites are a plague on the human condition in many developing countries with limited health care and sanitation. The morbidity produced by these parasites limits human health, development and prosperity. Nematode parasites also adversely affect animal welfare and production. Vaccines are not effective, so anthelmintic drugs are necessary for prophylaxis and treatment. Most anthelmintics belong to one of three classes: the macrocyclic lactones (ivermectin, moxidectin); the nicotinic anthelmintics (levamisole, pyrantel, derquantel) or; the benzimidazoles (albendazole, mebendazole). With the limited number of drugs available, there is real concern about the development of resistance. Tribendimidine was developed in China in the mid-1980s as a broad spectrum anthelmintic against soil-transmitted nematodes. Its mode of action has been investigated molecularly in *C. elegans* and on expressed nAChRs but, its mode of action has not been investigated directly in parasitic nematodes. Here we describe its effects on muscle contraction and electrophysiology in the pig nematode parasite, *A. suum*, which is very similar or the same as the human parasite, *A. lumbricoides*. Here we show that tribendimidine is a B-subtype selective nicotinic anthelmintic agonist that activates muscle nAChRs that are pharmacologically different from other cholinergic anthelmintics. It is concluded that tribendimidine could be effective against nematode parasites resistant to another cholinergic anthelmintic.

## Introduction

**Fig 1:**
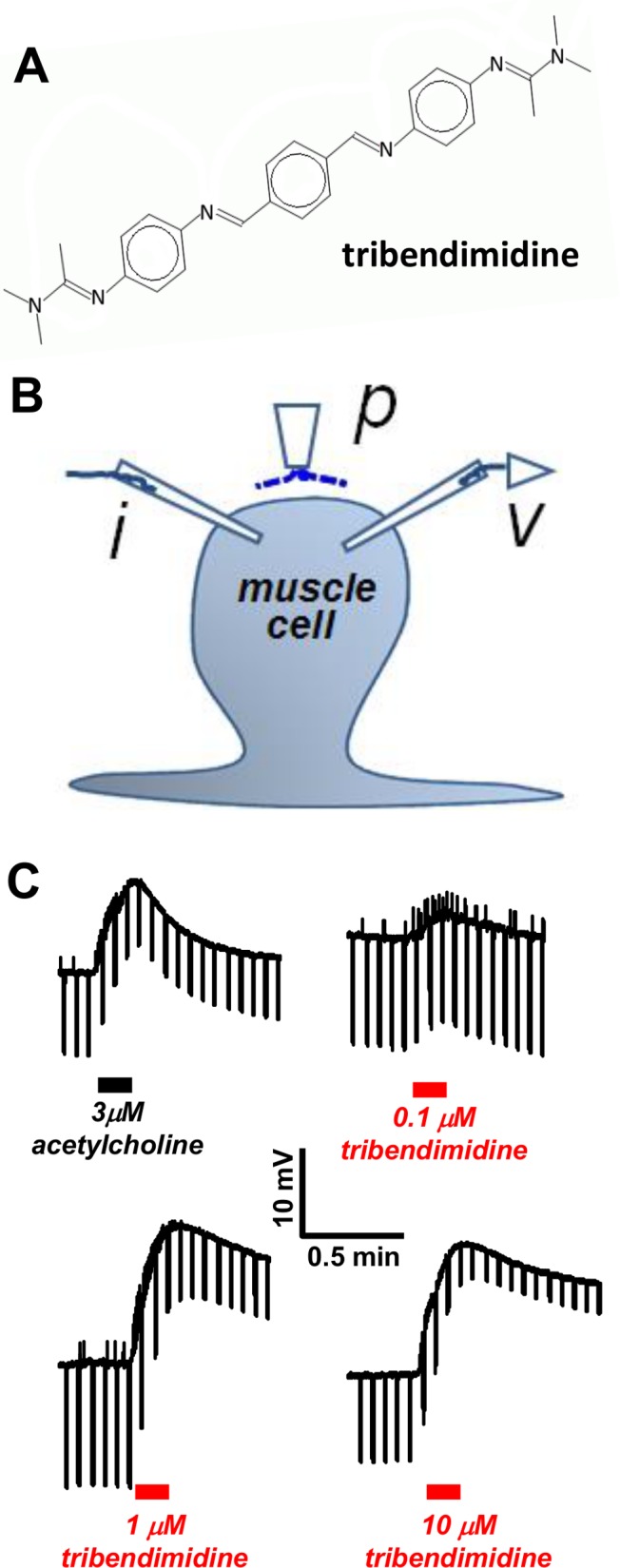
A: Chemical structure of tribendimidine. **B:** Diagram of the two-micropipette current-clamp technique used to record the membrane potential (*v*) and to inject (*i*) 40 nA hyperpolarizing 500 ms current pulses at 0.3 Hz. P is the microperfusion pipette used to apply and wash off the drugs. **C:** Application of 3 μM acetylcholine and then 0.1, 1 and 10 μM tribendimidine in the same preparation. Notice that 1 μM tribendimidine produces a bigger depolarization response (upward movement) and conductance increase (reduction in the voltage responses to current injection, producing a narrowing of the width of the trace) than 3 μM acetylcholine.

Limiting the debilitating effects of **S**oil-**T**ransmitted **H**elminth (**STH**) infections in humans and animals is a challenge. Effective vaccines are not available, and sanitation and clean water are not universally available. de Silva *et al*. [1] estimated that: there are 1.24 billion people infected with *A. lumbricoides*; there are 811 million people infected with *Trichuriasis*; and 716 million people are infected with hookworm. There are only a limited number of anthelmintic drugs available for human treatment [2]. On the World Health Organization list of essential medicines, there are four anthelmintics for treatment of soil transmitted nematodes: the benzimidazoles, albendazole and mebendazole; and the nicotinic agonists, pyrantel and levamisole. This list needs to be expanded and one additional drug may be tribendimidine. Tribendimidine has a symmetrical diamidine structure (Fig. 1A) that was developed by the Chinese CDC in the mid-1980s and the China State FDA approved it for human use in 2004 [3]. It has a broad-spectrum of action when used in a single-dose against parasitic nematodes of humans: it is effective against hookworm, *Ascaris, Strongyloides* but not *Trichuris* [4,5]. It also has effects again flatworm [5] and a potential for single-dose **M**ass **D**rug **A**dministration (MDA). However, its mechanism of action in nematode parasites has not been fully characterized.

Little was known of its mode of action until Hu *et al*. [6] published observations on *C. elegans*. These authors used null mutants and showed that for tribendimidine to immobilize *C. elegans* levamisole, L-nAChRs were required. These experiments are helpful but limited because of concerns that the levamisole receptor of *C. elegans* has different pharmacological properties to the muscle receptors of parasitic nematodes. The *C. elegans* L-nAChR is a single pharmacological receptor subtype which is not activated by nicotine. Nematode parasite levamisole nAChRs receptors are, however, activated by both nicotine and levamisole and they are a mixture of pharmacological subtypes of receptors. The *C. elegans* levamisole-nAChR is a pentameric receptor composed of UNC-38:UNC-29:UNC-63:LEV-1:LEV-8 subunits [7]. The parasitic nematode receptors include a number of subtypes composed of different combinations of UNC-38:UNC-29:UNC-63:ACR-8 subunits [8,9]. The parasitic nematode *Ascaris suum* has three separable subtypes known as the ***N*-, *L***- and ***B***-subtypes [10,11,12]. The ***N***-subtype is selectively activated by nicotine and oxantel; the ***L***-subtype is selectively activated by levamisole and antagonized competitively by paraherquamide; the ***B***-subtype is selectively activated by bephenium and selectively antagonized by derquantel (= 2-desoxyparaherquamide) [10,11].

We hypothesized that tribendimidine is a potent muscle nAChR agonist in parasitic nematodes but that it may not be an L-subtype selective agonist like levamisole because it has a very different chemical structure to levamisole. The significance of determining the subtype selectivity for tribendimidine relates to whether or not tribendimidine could be active in worms that are resistant to other cholinergic anthelmintics like levamisole or pyrantel and if tribendimidine could be synergistic with levamisole [13]. Such information is important for managing resistance and knowing how to combine tribendimidine with other nicotinic anthelmintics

Here we test the hypothesis that tribendimidine is a cholinergic anthelmintic using electrophysiological experiments. We examine its nicotinic receptor subtype selectivity to find that it is more selective for the *B*-subtype but not selective for the same *L*-subtype of receptors as levamisole. Tribendimidine may therefore be effective when levamisole is not, and tribendimidine may not show cross-resistance with levamisole.

## Methods

### *Ascaris suum* preparations

Adult *A. suum* were obtained weekly from the JBS Swift pork packing plant at Marshalltown, Iowa and maintained in Locke’s solution at 32^°^C, changed daily and the worms were used within 3 days of collection for contraction experiments and within 5 days of collection for electrophysiology experiments.

### Electrophysiology

We prepared 1 cm muscle tissue flaps from the anterior 2–3 cm part of the worm that was then pinned onto a Sylgard^™^-lined double jacketed bath chamber maintained at 35°C by inner circulation of warm water. The preparation was continuously perfused, unless otherwise stated, with *Ascaris* Perienteric Fluid-Ringer (APF-Ringer) [composition (mM): NaCl 23, Na-acetate 110, KCl 24, CaCl_2_ 6, MgCl_2_ 5, glucose 11, and HEPES 5; adjusted the pH to 7.6 with NaOH]. The rate of perfusion was 3.5–4 ml/min through a 20 gauge needle placed directly above the muscle bag recorded from. The experimental compounds were dissolved in APF-Ringer.

A two-micropipette current-clamp technique was employed to examine the electrophysiological effects in the bag region of *A. suum* muscle (Fig. 1B). We used 3M potassium acetate in the micropipettes with resistances of 20–30 MΩ. The recordings were obtained by impaling the bag region of *A. suum* muscle with 2 microelectrodes, one for current injection (*I*) and one for voltage recording (*V*). All experiments were performed using an Axoclamp 2A amplifier, a 1320A Digidata interface and Clampex 9 software (Molecular Devices, CA, USA) displayed and analyzed on a PC based desktop computer.

The current injecting electrode injected 500ms 40 nA hyperpolarizing step currents at 0.3 Hz, while the voltage recording electrode recorded the change in membrane potential in response to the injected currents. Cells with constant membrane potentials more negative than -20 mV for 20 min and a stable input conductance of < 3.5 μS were selected for the recordings.

### Contraction assays

Two 1-cm body-flap preparations, one dorsal and one ventral, were made from each *A. suum* female from the region anterior to the genital pore. Each flap was monitored isometrically on a force transducer in an experimental bath at 37°C containing 10 ml of the APF-Ringer bubbled with nitrogen. After dissection, the preparations were allowed to equilibrate for 15 min under an initial tension of 2.0 g. The antagonist was then added to the preparation 15 min before the application of the first concentration of the agonist. The agonists were added cumulatively with 2–3 min intervals between applications and the responses were steady changes in tension in response to increasing tribendimidine concentrations. The responses for each concentration were measured as the gram force tension produced and also expressed as the % of the maximum contraction.

Changes in isometric muscle tension responses were monitored using a PowerLab System (AD Instruments Colorado Springs, CO) that consists of the PowerLab hardware unit and Chart for Windows software. Sigmoid dose-response curves for each individual flap preparation at each concentration of antagonist were fitted using Prism 5.01 (GraphPad Software, San Diego, CA, USA) to estimate the constants by non-linear regression for each group of preparations receiving the same treatment. In preparations where desensitization was evident, the maximum response was used for fitting. The *pEC*_*50*_ was calculated as the negative logarithm of *EC*_*50*_. The agonist concentration-response relationship at each concentration of antagonist were illustrated and described by the lines best fitted to the Hill equation (constants: *pEC*_*50*_; slope, *nH*; and maximum response). The *pA*_*2*_ was obtained by fitting the equation:

$$pEC_{50}=-log\left({\left[X_{B}\right]}^{N}{+}_{}10^{-pA2*N}\right)—logC.$$

Where *pEC*_*50*_ is the same as before, *X*_*B*_ is the concentration of the antagonist, *N* is the equivalent to the slope of the Schild plot, *pA*_*2*_ is the concentration of the antagonist producing a dose-ration of 2 and C is a constant (-*log C* is the difference between [*pA*_*2*_ X *N]* and the agonist control curve *pEC*_*50*_. The *pA*_*2*_ estimates for methyllycaconitine, paraherquamide and derquantel with tribendimidine were made were made using the Prism software. The estimation of the *pA*_*2*_ rather than *pK*_*B*_ is appropriate because the value of *N* for the competitive model for each agonist-antagonist pair is sometime less than unity and the distribution of the data is log-normal [12].

Cluster analysis was conducted on the *pA*_*2*_ values obtained with the antagonists, methyllycaconitine, paraherquamide and derquantel for the agonist tribendimidine and those that had previously be determined using the same methods for bephenium, thenium, pyrantel, levamisole, oxantel, methyridine and nicotine [12]. We used Minitab 13.2 (State College, PA) for the cluster analysis (average, squared Euclidian, standardized variable) to determine the similarity. Fuller details of the contraction assay, use of *pA*_*2*_ and methods are available [12]

### Larval migration studies

Levamisole-sensitive and levamisole-resistant *O. dentatum* were originally supplied by the Royal Veterinary and Agricultural School, Frederiksberg, Copenhagen and then reproduced at yearly intervals by passage in pigs at Iowa State University, Ames, Iowa. The *L*_*3*_ isolates were maintained between passages in tap water refrigerated at 11°C. The levamisole-resistant isolates were maintained under selection pressure by collecting eggs for *L*_*3*_ preparation following treatment with a therapeutic dose of 8 mg/Kg levamisole. The pigs were infected and following collection of the parasites killed humanely: all animal procedures complied with and were governed by Institutional Animal Care and Use Committee (IACUC) regulations under U.S. Federal laws and policies which required formal written prior approval by the IACUC committee of the procedures as well as veterinary supervision of the animals. 1,500–3,000 *L*_*3*_s were ex-sheathed by 5–10 min incubation in 1.5% sodium hypochlorite solution. The larvae were then washed 3 times in migration buffer (composition: 0.85% NaCl, 5 mM Tris-HCl, pH to 7.0 with 1 M NaOH) with the help of centrifugation (5 min at 500 *g*). 150 larvae were collected with a pipette and placed in each of the drug concentrations to be tested for 2 h at 37°C. After incubation, *L*_*3*_ larvae were re-suspended in fresh test solutions. The migration apparatus was made of two tightly fitting plastic tubes (∼ 10 mm length) secured to a 20 μm nylon filter placed in each test solution of a 24-well plate. The re-suspended larvae were added to the top of each filter, allowed to migrate through the filters and into the wells during 2 h incubation at 37°C. At the end of the incubation period, the number of larvae remaining within each of the filter tubes was counted and the number of larvae entering into the 24 well plates was counted. We then calculated the percentage of larvae not migrating for each of the concentrations. The relationship between the concentrations of levamisole and the percentage of inhibited larvae was then examined by fitting the Hill equation to describe the sigmoidal dose-response curves. The relationship between the concentrations of tribendimidine is the percentage inhibited larvae was examined in the same way.

### Drugs

Tribendimidine was a gift of Prof S.H. Xiao, National Institute of Parasitic Diseases, Shanghai, and Peoples Republic of China. Derquantel and paraherquamide, a gift of Pfizer Animal Health (now Zoetis) were dissolved in DMSO and used at a maximum concentration of 0.1%. All other drugs were obtained from Sigma-Aldrich, St. Louis, MO. Tribendimidine was initially dissolved in DMSO and diluted in APF to the concentrations shown in results. The maximum concentration of DMSO used was 0.1%, a concentration that was tested and found to have no effect. The maximum soluble concentration of tribendimidine that was used was 30 μM.

## Results

### Electrophysiology shows that tribendimidine is a nicotinic agonist on *Ascaris* muscle

Tribendimidine (Fig. 1A) is a derivative of amidantel which is a cholinergic anthelmintic. Molecular studies in *C. elegans* [6] suggests that tribendimidine acts selectively as an agonist, like levamisole, on nematode muscle nicotinic receptor ion-channels. To test the hypothesis that tribendimidine is a cholinergic agonist on parasite muscle, we used a two-micropipette current-clamp technique (Fig. 1B) to record from *A. suum* somatic muscle during microperfusion of the acetylcholine and tribendimidine. We observed that tribendimidine produced a concentration-dependent and reversible depolarization associated with an increase in membrane conductance like acetylcholine (Fig. 1C) and other cholinergic anthelmintics in *A. suum* [14]. Tribendimidine is more potent than acetylcholine and this is also illustrated in Fig. 1C which shows a recording of the effects of acetylcholine (1 μM) and tribendimidine (0.1, 1 and 10 μM) from the same muscle cell: the depolarization effect (10.7 mV) of 3 μM acetylcholine was less than the depolarizing effect (14.3 mV) of 1 μM tribendimidine.

**Fig 2:**
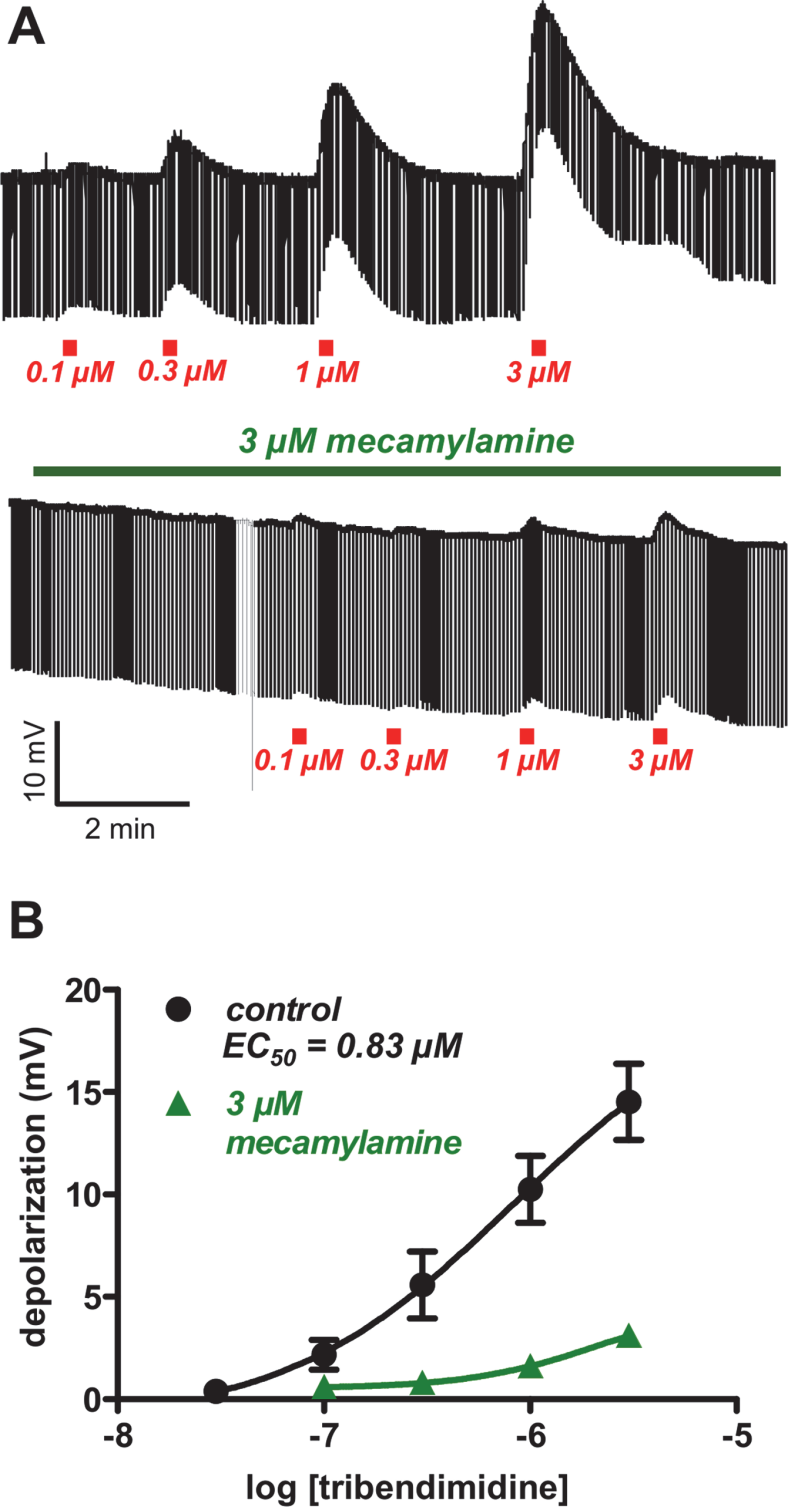
A: Top, representative recording of the application, for 10 seconds, of different concentrations of tribendimidine which produces a depolarization (upward movement of the thickest line) and increase in membrane conductance (decrease in the width of the trace). Bottom, representative recording of the application for 10 seconds, of tribendimidine in the presence of 3 μM mecamylamine, a nicotinic antagonist, with inhibition of the tribendimidine responses. **B:** Plots of the mean ± s.e. (n = 4) of the tribendimidine-concentration-depolarization-responses in the absence and presence of 3 μM mecamylamine. The control plot was fitted to the Hill equation allowing estimation of the control *EC*_*50*_ as 0.83 μM.

To determine the concentration-response characteristics of tribendimidine, ten-second applications of tribendimidine were used at different concentrations at, and greater than 0.03 μM (Fig. 2A). The concentration-depolarization response plot had an *EC*_*50*_ of 0.83 μM (log *EC*_*50*_ = -6.08 ± 0.67; n = 36, Fig. 2B). Mecamylamine is a potent nicotinic antagonist on *Ascaris* muscle acetylcholine receptors, which at a concentration of 3 μM also non-competitively antagonized the effects of tribendimidine on membrane potential (Fig. 2A & B). These observations are consistent with tribendimidine acting as a potent cholinergic anthelmintic agonist on *Ascaris* muscle.

### Acetylcholine subtype selectivity of tribendimidine

**Fig 3:**
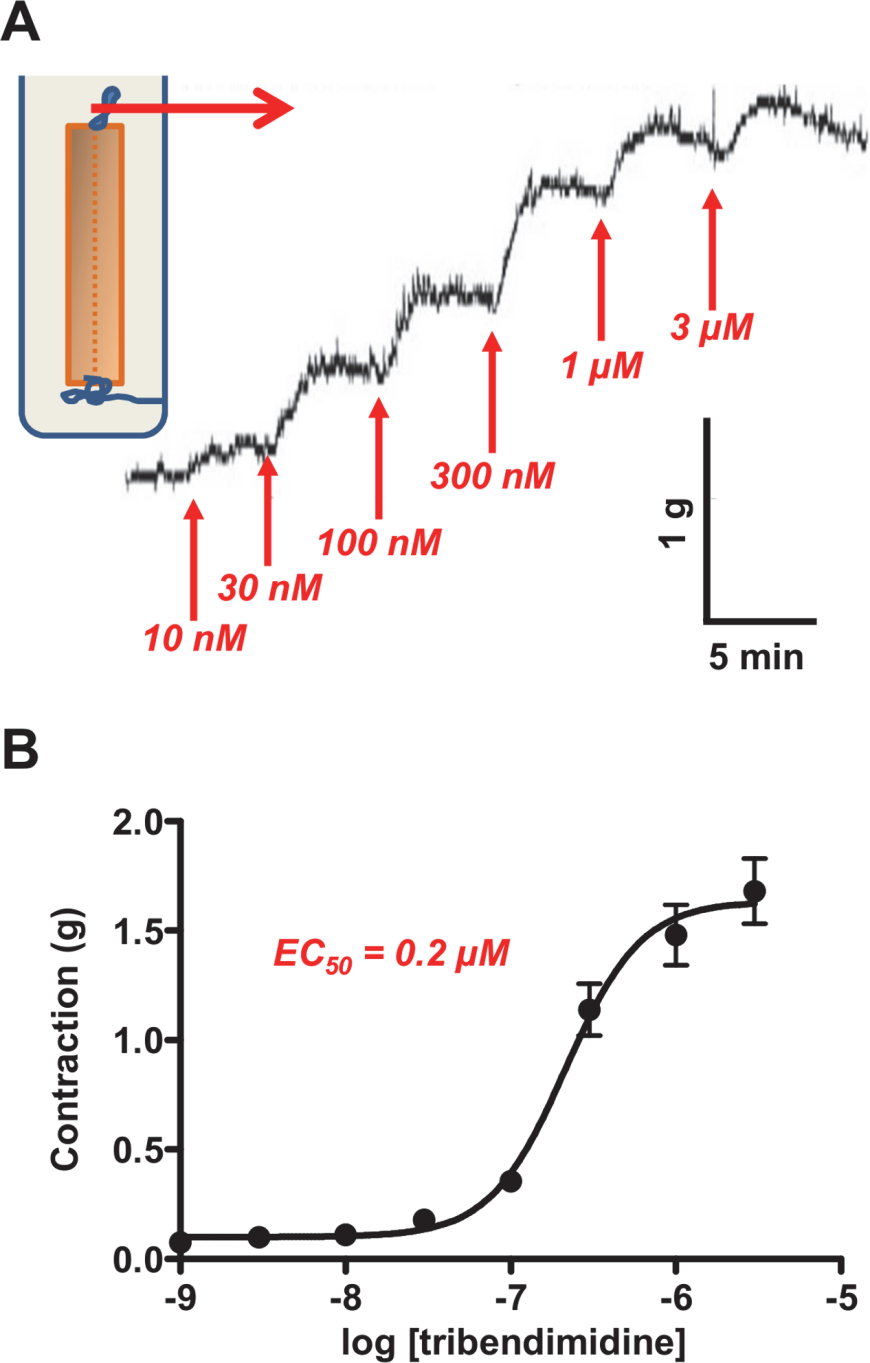
Contraction of *Ascaris suum* muscle strips produced by tribendimidine. **A:** Diagram of the isometric contraction technique used to measure the force of contraction and representative trace of a cumulative-tribendimidine-contraction-response. **B:** Plot of the mean ± s.e. (n = 6 or more) cumulative-tribendimidine-contraction-response and Hill equation fit with and *EC*_*50*_ of 0.2 μM. The *EC*_*50*_ for contraction is, as expected, is lower than for the membrane potential response because the amplification in the depolarization-contraction signaling cascade of muscle.

The nicotinic cholinergic receptors on *A. suum* muscle have been separated into three different subtypes based on their pharmacology in muscle contraction experiments and characterized by their single channel conductance in patch-clamp experiments [11,12]. The different types appear to be due to different arrangements of the subunits of the nicotinic receptor [8,9]. We used the *Ascaris* muscle strip preparation (Fig. 3A) along with the competitive antagonists derquantel, paraherquamide and methyllycaconitine to examine the subtype selectivity of tribendimidine [12]. The principle of the technique is to measure the *pA*_*2*_s (antagonist potencies) of the three antagonists against tribendimidine using the competitive antagonism model and to compare the *pA*_*2*_ values against the *pA*_*2*_ obtained for other cholinergic anthelmintics. We used cluster analysis to pharmacologically group the selectivity of tribendimidine.

**Fig 4:**
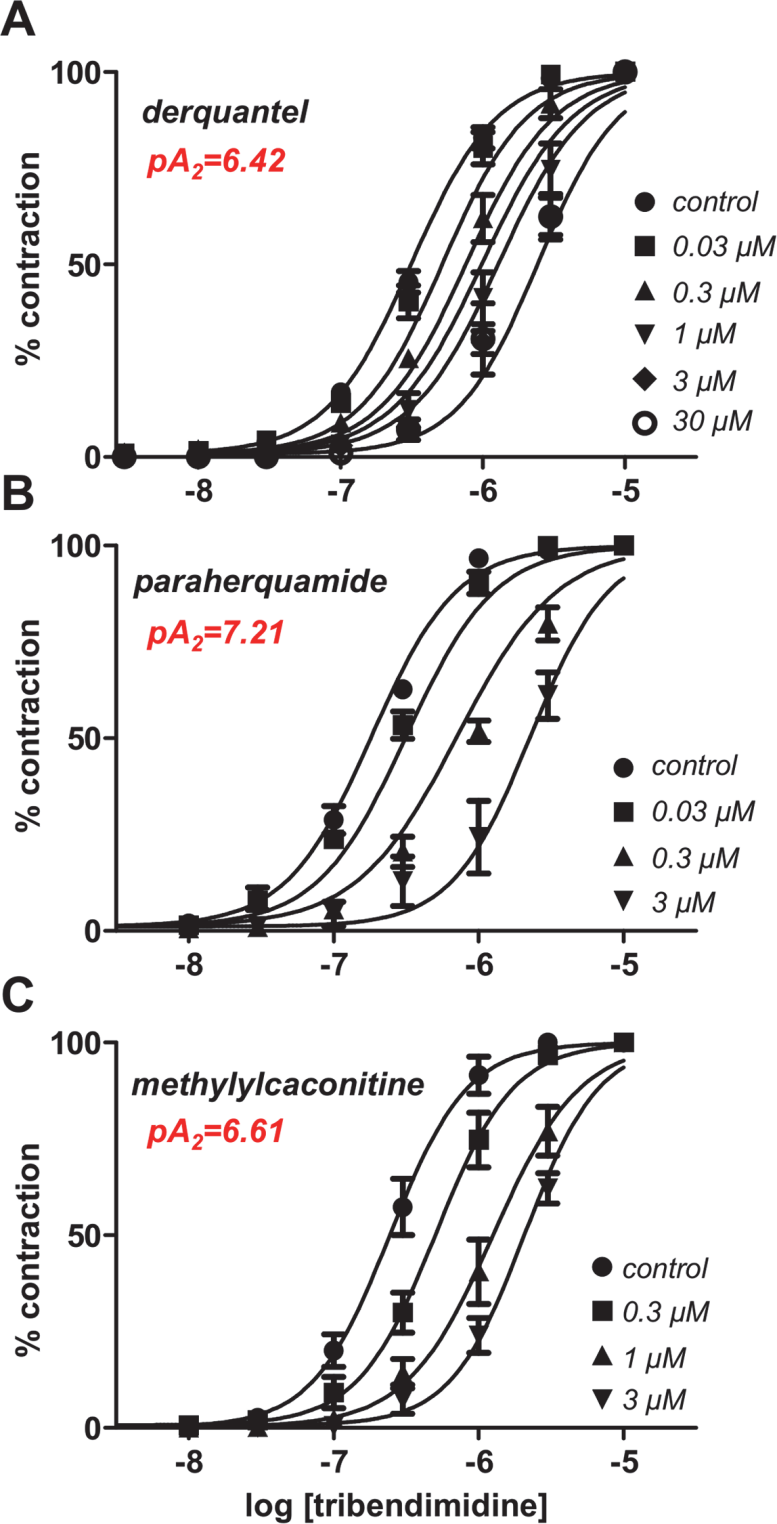
Effect of antagonists of the tribendimidine-concentration-contraction plots allowing the estimation of *pA*_*2*_ values. **A:** Effect of derquantel *pA*_*2*_ = 6.42 ± 0.12 mean ± s.e. (n = 108). **B:** Effect of paraherquamide *pA*_*2*_ = 7.21 ± 0.13 mean ± s.e. (n = 90). **C:** Effect of methyllycaconitine (MLA) *pA*_*2*_ = 6.61 ±0.09 mean ± s.e. (n = 90).

**Fig 5:**
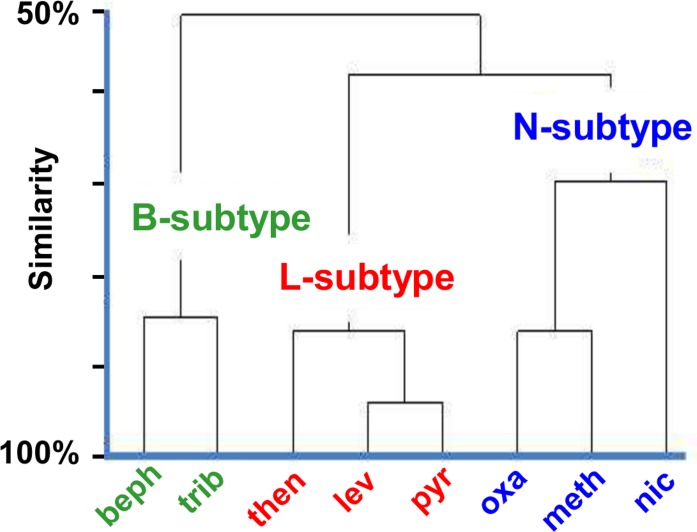
A: Similarity Cluster analysis of *pA*_*2*_s of the antagonists, derquantel, paraherquamide and methyllycaconitine against the agonists beph (bephenium), trib (tribendimidine), then (thenium), lev (levamisole), pyr (pyrantel), oxa (oxantel), meth (methyridine), nic (nicotine) (ordinate: similarity, averaged, squared Euclidian, standardized variable, estimated using Minitab 13.2 (State College, PA) showing that tribendimidine does not group with levamisole and is closer to bephenium. **B:** Schematic illustration of the selectivity of tribendimidine and levamisole for B-subtype (green) and L-subtype (red) nAChRs.

Fig. 3A shows a representative tribendimidine cumulative-concentration contraction-response experiment for an *Ascaris* muscle strip and Fig. 3B shows the plot of means ± s.e. (n ≥ 4) for the tribendimidine responses. Tribendimidine had a potent effect on contraction and the *EC*_*50*_ for tribendimidine contraction was 0.2 μM. We tested the effects of different concentrations of derquantel (Fig. 4A) paraherquamide (Fig. 4B) and methyllycaconitine (Fig. 4C) on the tribendimidine responses. Each concentration-response was fitted with the modified Hill Equation restrained to be parallel to yield estimates of the *pA*_*2*_s of the antagonists with tribendimidine as the agonist. The *pA*_*2*_ for derquantel was 6.42±0.15 (377 nM); the *pA*_*2*_ for paraherquamide was 7.21±0.13 (62 nM); and the *pA*_*2*_ for methyllycaconitine was 6.61±0.09 (219 nM). Cluster analysis of these values was used to compare these *pA*_*2*_ values with the cholinergic anthelmintic agonists, bephenium, thenium, levamisole, pyrantel, oxantel, methyridine and nicotine [12]. Fig. 5 shows that tribendimidine is most similar to and clusters with bephenium (B-subtype) and not with levamisole (L-subtype) or nicotine (N-subtype), suggesting that it has similarities to bephenium and has a different subtype selectivity to levamisole, and nicotine.

### Tribendimidine remains effective against levamisole-resistant larvae

**Fig 6:**
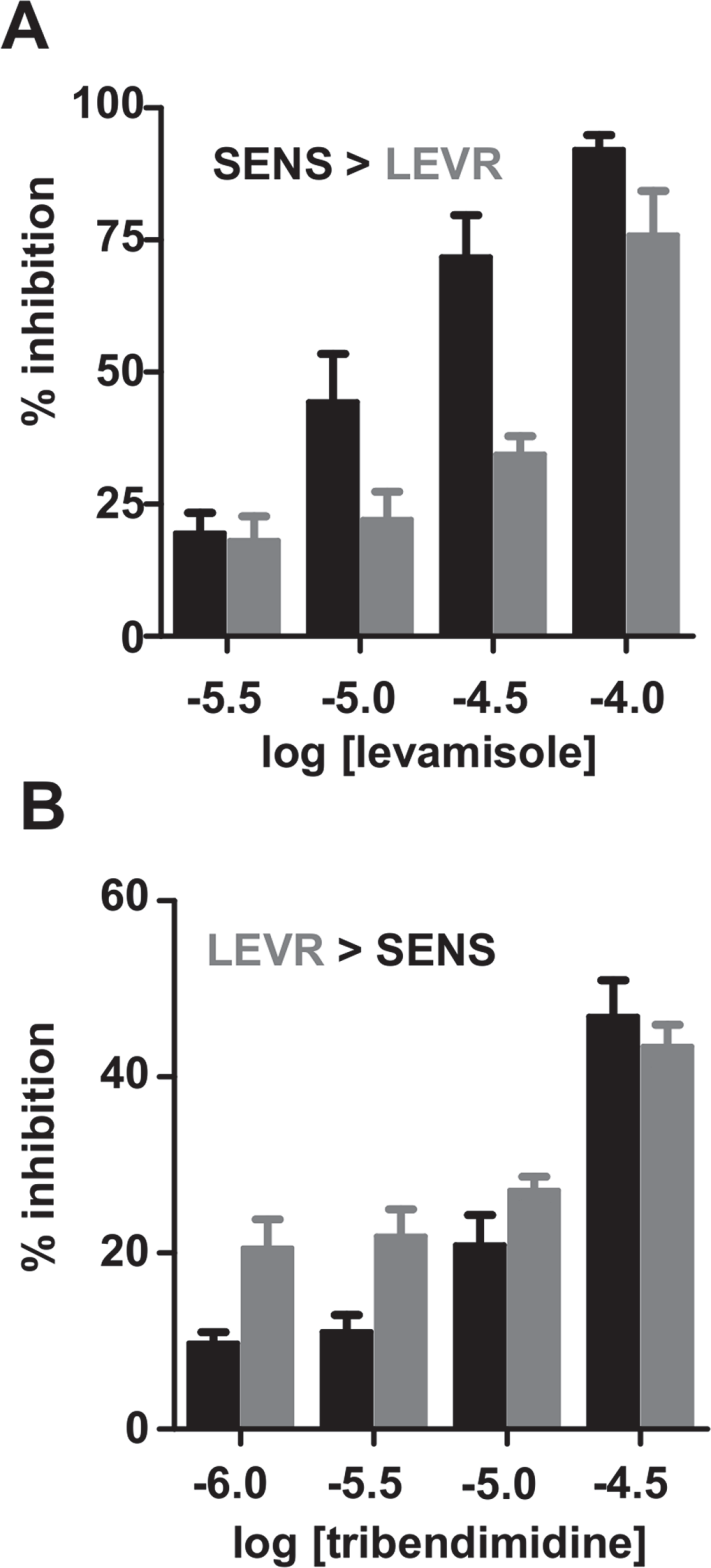
Larval migration inhibition assays on *O. dentatum L*_*3*_ levamisole-sensitive (SENS) and levamisole-resistant (LEVR) isolates. **A:** Levamisole is less effective on levamisole-resistant isolates than on the levamisole-sensitive isolates and the difference is significant (p<0.001, F-test). **B**: tribendimidine is more potent on levamisole-resistant isolates than on levamisole-sensitive isolates and the difference is significant (p<0.001, F-test).

We tested the effects of levamisole and tribendimidine on larval motility using the larval migration inhibition assay with *Oesophagostomum dentatum L*_*3*_ larvae (Fig. 6) on levamisole-sensitive and levamisole-resistant isolates [14]. Levamisole (Fig. 6A) was more potent (p< 0.001, F-test) at inhibiting the migration of SENS (levamisole-sensitive larvae) than the migration of LEVR (levamisole-resistant) larvae. We tested the effects of tribendimidine to the limits of its solubility (∼30 μM), Fig. 6B, and found that tribendimidine was more potent on the levamisole-resistant isolate (LEVR) than on the levamisole-sensitive (SENS) isolate and that the difference was significant (p < 0.001, F-test). The bigger effect of tribendimidine on the LEVR than the SENS isolate suggests that these two drugs do not activate the same nAChR receptor subtypes.

## Discussion

### *C. elegans* and tribendimidine

Hu *et al.*, [6] have described how, in *C. elegans*, null-mutants of 11 genes (including the subunit genes: *unc-63; unc-38, unc-29, lev-1* and *lev-8*) produce both levamisole-resistance and tribendimidine-resistance. Their observations suggest that tribendimine activates levamisole (L-type) receptors like levamisole and pyrantel. When we compare these observations on levamisole and tribendimidine in *C. elegans* with our observations on parasitic nematodes, there is a difference: the *C. elegans* observations suggest that tribendimidine acts selectively and exclusively on its L-type nAChRs; but our observations with parasitic nematode suggest that tribendimidine activates nAChRs that levamisole does not. The levamisole receptor of *C. elegans* is made of an obligatory pentameric arrangement of UNC-63, UNC-38, LEV-8, LEV-1 and UNC-29; omission of one of these subunits will cause levamisole and tribendimidine resistance [6] and prevent expression in *Xenopus* oocytes [6,7]. Thus loss of one subunit in *C. elegans* will lead to resistance of both levamisole and tribendimidine because only one type of receptor is present.

### In parasitic nematodes tribendimidine is less selective for the L-subtype

In the parasitic nematodes *A. suum* and *O. dentatum* [11,15] there are heterogeneous muscle nAChRs subtypes. Although receptor subtypes present in *A. suum* muscle strips may not all be the same as those present in *O. dentatum* larvae, particularly since *A. suum* is Clade III and *O. dentatum* is Clade V suggesting that these nematode parasites are very well separated evolutionarily and molecularly, there is evidence of the presence of an L-subtype in both species [8,11,15] and other subtypes. The receptor pool subtypes of muscle nAChR can be produced by different combinations of the receptor subunits [8,9,16]. In *Ascaris*, we can separate three pharmacological subtypes: the N-subtype which is more sensitive to nicotine; the B-subtype which is more sensitive to bephenium; and the L-subtype which is more sensitive to levamisole. In adult *Oesophagostomum* there is also evidence of the L-subtype of nAChR [8,15] but the other subtypes have not yet been separated pharmacologically

Our cluster analysis of the *Ascaris* resulted shows that tribendimidine is more pharmacologically similar to bephenium than levamisole and is more selective for the B-subtype of nAChR subtype than levamisole (Fig. 5). The different selectivity of tribendimidine for the B-subtypes and of levamisole for the L-subtypes may explain differences in the effects of levamisole and tribendimidine on levamisole-sensitive and levamisole-resistant larvae in the larval migration studies. The levamisole-sensitive larvae (SENS) appear to have more L-subtype receptors present than the other subtypes because levamisole is more potent than tribendimine. With the levamisole-resistant isolate (LEVR), a loss of L-subtype receptors could lead to a reduction in the levamisole sensitivity and an apparent increase in the other nAChR receptor subtypes.

### Anthelmintic potential

We have observed that tribendimidine will produce depolarization (*EC*_*50*_ = 0.8 μM) of *Ascaris* muscle and contraction of *Ascaris* muscle strips in a concentration-dependent manner (*EC*_*50*_ = 0.2 μM) with B-subtype selectivity. A different subtype selectivity for tribendimidine and levamisole on nAChR receptors subtypes has also been observed for *O. dentatum* expressed receptors [8] supporting the notion that tribendimidine has the potential to be effective against nematode parasites that are not sensitive to levamisole. Tribendimidine then, has an interesting and promising pharmacology and has potential for single-dose MDA with its broad-spectrum. Its future use as an effective broad-spectrum anthelmintic against soil-transmitted helminths and flatworm [5], could be compromised because of its metabolism to p-(1-dimethylamino ethylimino) aniline and terephthalaldehyde [17,18,19] which are dimethylamino anilines that are potential carcinogens and mutagens [20,21]; but these metabolites are completely broken down and eliminated within 24 h reducing the chances of carcinogenic effects. Although tribendimidine appears safe and has broad-spectrum, a large-scale clinical study is advocated to further verify safety [5].

### Conclusion

The contraction and electrophysiological effects of tribendimidine demonstrate a nicotinic agonist action on nAChRs present on *Ascaris* muscle. Tribendimidine has a selective effect on the B-subtype of nAChRs rather than the L-subtype of nAChR as in *C. elegans*. The observations highlight the need to examine anthelmintic modes of action in parasitic nematodes as well as *C. elegans* because of their possible differences. Finally our observations suggest that tribendimidine may be useful for soil-transmitted nematodes that are not susceptible to levamisole.
